# Third body damage and wear in arthroplasty bearing materials: A review of laboratory methods

**DOI:** 10.1016/j.bbiosy.2021.100028

**Published:** 2021-09-06

**Authors:** Raelene M Cowie, Louise M Jennings

**Affiliations:** Institute of Medical and Biological Engineering, School of Mechanical Engineering, University of Leeds, Leeds LS2 9JT, UK

**Keywords:** Arthroplasty, Implant, Third body wear, Experimental simulation, Polyethylene

## Abstract

Third body wear of arthroplasty bearing materials can occur when hard particles such as bone, bone cement or metal particles become trapped between the articulating surfaces. This can accelerate overall implant wear, potentially leading to early failure. With the development of novel bearing materials and coatings, there is a need to develop and standardise test methods which reflect third body damage seen on retrieved implants. Many different protocols and approaches have been developed to replicate third body wear in the laboratory but there is currently no consensus as to the optimal method for simulating this wear mode, hence the need to better understand existing methods. The aim of this study was to review published methods for experimental simulation of third body wear of arthroplasty bearing materials, to discuss the advantages and limitations of different approaches, the variables to be considered when designing a method and to highlight gaps in the current literature. The methods were divided into those which introduced abrasive particles into the articulating surfaces of the joint and those whereby third body damage is created directly to the articulating surfaces. However, it was found that there are a number of parameters, for example the influence of particle size on wear, which are not yet fully understood. The study concluded that the chosen method or combination of methods used should primarily be informed by the research question to be answered and risk analysis of the device.

## Highlights

• Review of the published methods for experimental simulation of third body wear of arthroplasty bearing materials.
• Discussion of the advantages and limitations of different approaches for third body wear simulation.
• Identification of variables to be considered when designing a third body wear simulation protocol and highlighting gaps in the current literature.

## 1 Introduction

Total joint replacement is one of the most common and successful surgical procedures carried out with over 200,000 new primary total hip (THR) and knee replacements (TKR) listed in the National Joint Registry Annual report for 2020 and an anticipated survivorship in excess of 90% at 10 years [1]. Despite advances in materials, implant wear and wear debris induced osteolysis leading to aseptic loosening remain causes of revision [1,2]. Wear can be determined pre-clinically in the laboratory using joint simulators, but as the demographic for patients receiving a joint replacement shifts to include those who are younger, more active or potentially obese, the demands on the implant increase. Thus preclinical evaluation should reflect this wider range of conditions [3].

**Fig. 1:**
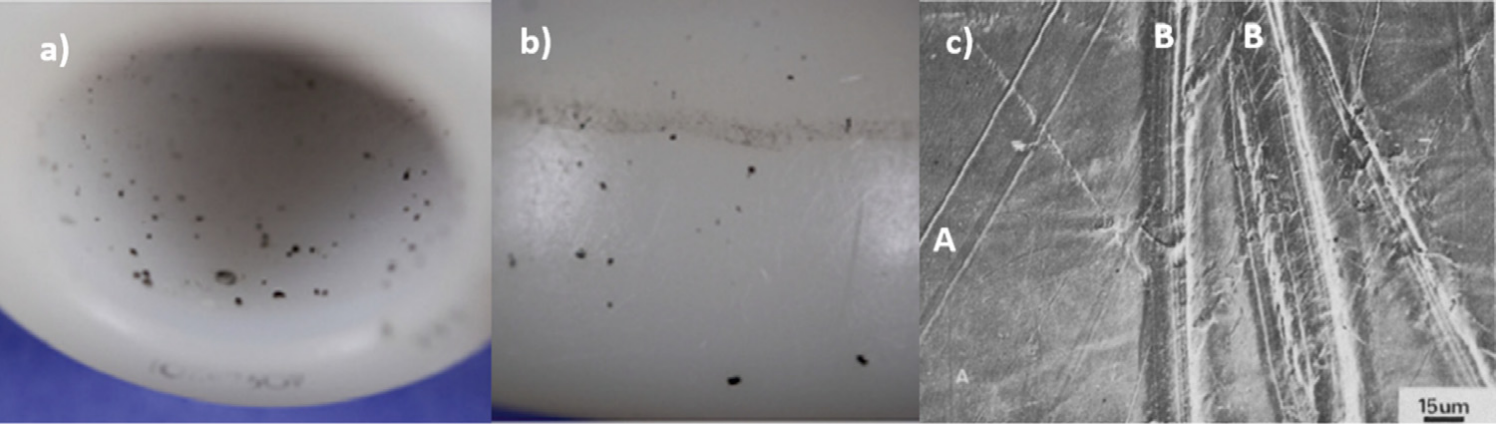
Polyethylene liner from a retrieved dual mobility hip with third body particles visible embedded in the polyethylene inner (a) and outer (b) bearing, adapted from Spece et al. [26]; c) shows an SEM image of a retrieved polyethylene cup, the fine scratches in region a are associated with surface wear, the deeper scratches (B) are thought to be caused by third body particles, adapted from Atkinson et al. [24].

**Fig. 2:**
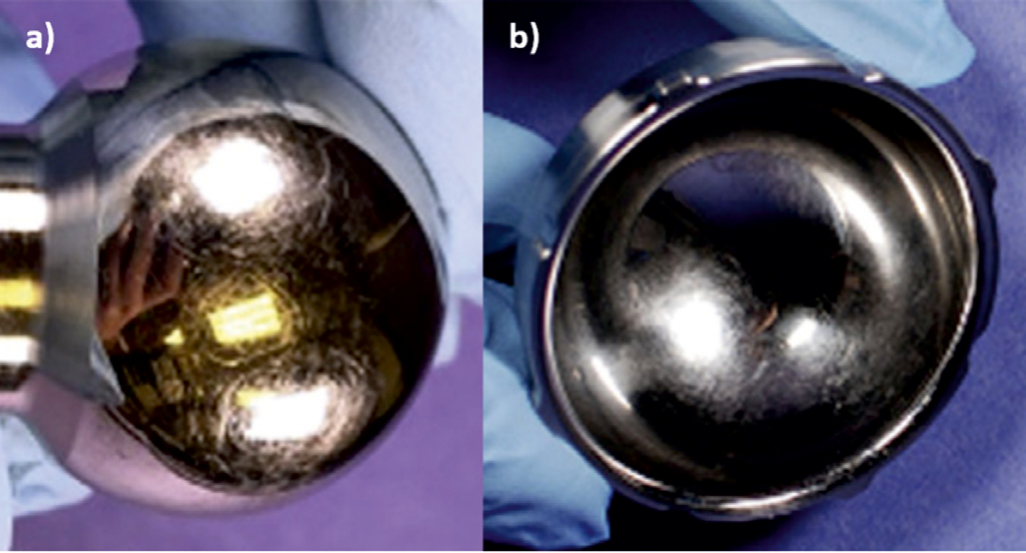
(a) scuffed head and (b) cup from a retrieved dual mobility hip with third body particles visible embedded in the polyethylene bearing, adapted from Spece et al. [26].

With current implant materials, for optimally positioned components simulated without contaminants, surface wear dominates [4,5] and methods have been standardised to replicate this wear mode in the hip, knee and ankle in laboratory simulation of a walking gait cycle [6], [7], [8], [9], [10]. However, the limited range of conditions specified in the international standards may not fully reflect the *in vivo* scenario or the damage identified on retrieved implants. Therefore, there is a need to further develop methods to replicate explant damage [11,12] and the activities undertaken by patients. [13] One wear mode that has been investigated experimentally but not yet standardized is third body wear. Third body wear can occur when hard particles such as bone, bone cement or metallic particles, become trapped between the articulating surfaces of a joint replacement, creating damage and accelerating implant wear [4,5,14]. Evidence of third body wear has been seen in retrieved hip [14], [15], [16], [17], [18] and knee implants [19], [20], [21], [22], particularly in metal-on-polyethylene bearing couples where particles such as polymethylmethacrylate (PMMA) cement, bone and porous coating beads have been found embedded in polyethylene and deformation or ploughing of the polyethylene [23] has been observed (Fig. 1) [17,20,[24], [25], [26]]. Scratching on metallic counterfaces understood to be caused by third body particles trapped between the articulating surfaces has also been identified (Fig. 2) [17,26,27]. For counterfaces to be scratched, the scratching material, in this case a third body particle, must be harder than that of the counterface [15,28]. If scratches in the implant surface are of sufficient magnitude, there is the potential to accelerate implant wear. The relative hardness of the particles and substrate are therefore an important factor in determining the abrasion resistance of an implant. Studies have suggested that if the hardness of the substrate is less than 0.8 of the third body particles present in the joint, severe abrasion is likely to occur; when the hardness of the implant is greater than 1.2 times that of the third bodies, the implant can be considered resistant to a third body wear mode [29]. Therefore, third body wear could potentially be avoided by using bearing materials highly resistant to third body wear (such as composite ceramics currently used in hip replacements) [23] or by minimizing the presence of third body particles in the joint as much as possible by better operative procedures.

**Fig. 3:**
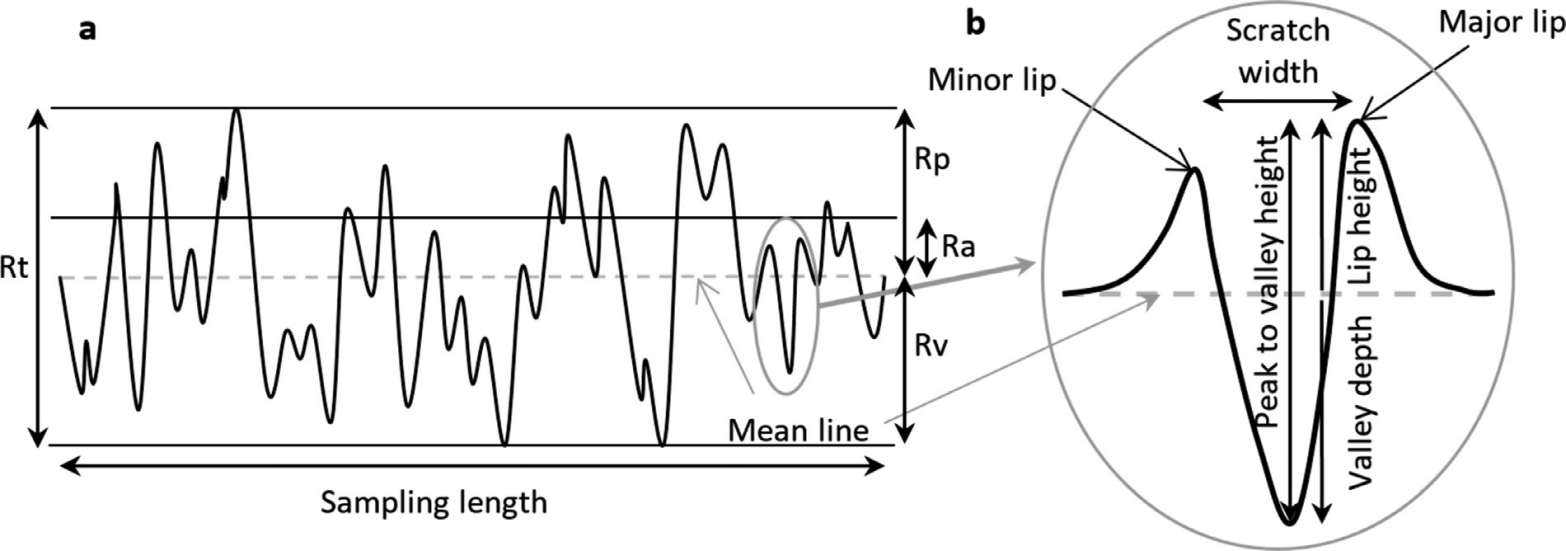
a:, Schematic showing 2D surface roughness parameters of interest for assessment of surfaces subjected to third body wear b: the morphology of a single scratch in a metal counterface with lips created by hard third body particles. [30, 34].

Ploughing of the metal surface by the third body particle may not directly lead to removal of material but the scratches generated may cause plastic deformation (cold flow) of the counterface forming ridges (lips) and grooves (valleys) and it is the lips of the scratches which may cause an abrasive wear mode to dominate, accelerating implant wear and subsequent failure [20,21,23]. The morphology of a single scratch may be characterized as shown in Fig. 3b, with the lip height of the scratch being the most significant parameter which influences wear. For surfaces containing multiple scratches, roughness indices which assess the surface topography of the implant over a large area may be more appropriate (Fig. 3a). The Ra (arithmetic average of the absolute values of the profile heights over the sampling length) gives a measure of the overall surface roughness whilst Rp (maximum profile height over a sampling length) and Rsk (skewness where a positive value indicates a greater percentage of the profile is above the mean line) better describe the extent to which material has been pushed above the mean line. For a metal surface, a higher Rp or more positive Rsk is more likely to lead to wear of an opposing polyethylene counterface [30]. Rv (maximum profile depth over the sampling length) and Rt (total profile height over the sampling length) are surface roughness parameters which may be used to further describe the articulating surfaces of a component. In more recent studies, equivalent areal parameters are often used *i.e*. Sa, Sp and Ssk. The severity and directionality of damage on retrieved metal femoral heads has been characterised in terms of ‘scratches’ and ‘scrapes’ using an image based computational technique. Thin, discrete damage was characterised as scratching, broad swathes of damage was termed scraping to give global characterization of the implants [27].

For metal counterfaces, an exponential relationship between lip height and polyethylene wear has been observed whereby the wear of polyethylene against scratches with a small lip height (< 0.5–1 µm depending on the polyethylene investigated) is similar to that of smooth counterfaces but above a threshold, there is a dramatic increase in polyethylene wear [31], [32], [33].

There are many potential sources of third body particles either from the implantation procedure, [35,36] from products used close to the articulating surfaces [37] or resulting from many years of implantation [5,38,39] and if released into the joint space, there is potential for these particles to become entrained between the articulating surfaces.

Extensive research was carried out into third body wear in the 1990′s and 2000′s alongside the development of materials for use in joint replacements. When interpreting historical data, it is important to understand not only the third body particles used but also the counterfaces of interest. For example, advances in the processing of polyethylene, including cross-linking, mean current commercially available total hip [40] and knee replacements [41] are generally considered to be more resistant to third body wear than conventional polyethylene [42]. Therefore when interpreting these studies, in particular with reference to implant wear, it is important to note not all the bearing materials discussed within this article are currently available for implantation. With the development of new implant materials and coatings, there is renewed interest in the third body wear mode. There is currently no consensus as to the optimum laboratory method for the pre-clinical experimental simulation of third body wear in total joint replacement hence the need to better understand existing methods. Therefore, the aim of this review is to assimilate and critique published methods previously used to simulate third body wear, and to inform method(s) that could be adopted as an international standard.

## 2 Methods for searching literature

### 2.1 Search strategy

A literature search was conducted to identify articles which carried out third body wear simulation on hip, knee or simple geometry using Google Scholar, PubMed and ScienceDirect in April 2021. Keywords used for the searches included combinations of, implant OR hip OR knee OR orthopaedics OR polyethylene AND third body wear OR third-body wear. The search was also expanded by reviewing the reference sections of selected papers to capture all relevant articles and searching online abstract books from the Orthopaedic Research Society annual meetings and International Society for Technology in Arthroplasty meetings.

### 2.2 Selection criteria

Only articles written in English and those which carried out third body wear simulation of joint replacements or materials used in joint replacement were included in the analysis. Duplicate publications and those where a full-text could not be retrieved were excluded.

### 2.3 Article selection

The article titles and abstracts were screened to determine their suitability for inclusion in the article. Full-text articles meeting the inclusion criteria were selected for data abstraction.

### 2.4 Data abstraction

Data was extracted from full text articles and abstracts and tabulated to compare each study, this supplementary information is available through the University of Leeds data repository [43]. 60 articles were identified in total published between 1987 and 2020. First, the articles were divided by the research method (simulating third body wear with particles or scratching the implant surface directly) then by the simulation system investigated (*i.e*. hip, knee, pin-on-plate/disc). For the studies carrying out third body wear with particles, the authors, year of study, information relating to the method including the materials of interest, particle type and diameter and whether single or multiple doses of particles were used, as well as the results including the presence of embedded particles, characterization of the surfaces and the wear of the materials. There were 17 articles identified relating to hip, 5 knee and 11 pin-on-plate/disc. For the studies where the surfaces of the implants were scratched directly, the author and year as well as the materials, details pertinent to the method, surface characterization and implant wear were extracted from the articles. In total there were 14 articles relating to hip, 6 knee and 10 pin-on-plate/disc. Some articles were duplicated as they used both particle and scratching methods.

## 3 Methods for simulation of third body wear

**Fig. 4:**
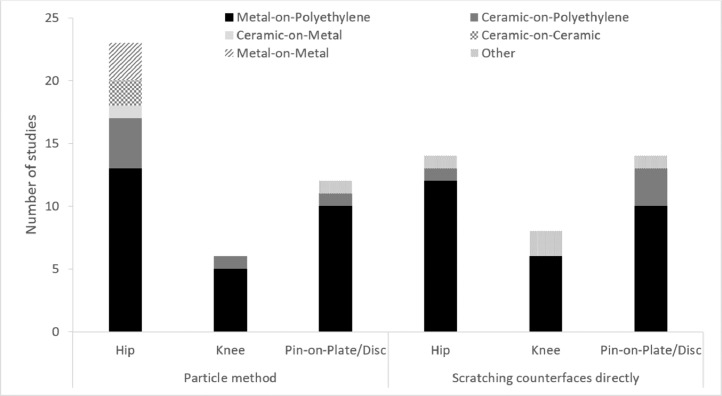
Number of studies using either a particle method or scratching the implant surfaces directly and the bearing material combinations used in these, where multiple materials have been investigated, both material combinations have been included, materials characterised as ‘other’ include bearing materials such as PEEK and oxidised zirconium. [15,23,28,29,[31], [32], [33],^42^,[44], [45], [46], [47], [48], [49], [50], [51], [52], [53], [54], [55], [56], [57], [58], [59], [60], [61], [62], [63], [64], [65], [66], [67], [68], [69], [70], [71], [72], [73], [74], [75], [76], [77], [78], [79], [80], [81], [82], [83], [84], [85], [86], [87], [88], [89], [90], [91], [92]].

Several approaches have been taken to simulate third body wear in total joint replacements, for the purposes of this review, the methods have been divided into two approaches. The first method uses clinically relevant particles introduced into the lubricant during wear simulation to replicate third body wear; the second approach involves directly roughening one of the articulating surfaces to create damage similar to that seen on retrieved implants, then carrying out wear simulation against the damaged surfaces. There are advantages and limitations to both approaches. The majority of studies have focussed on the wear of hip implants or have been carried out in simple geometry pin-on-plate or pin-on-disc configuration to investigate the articulating materials, studies into the wear of knee implants are less common (Fig. 4). Despite the potential for third body particles to enter the interface of any artificial joint, the most commonly investigated material combination is metal-on-polyethylene (Fig. 4).

### 3.1 Approach 1: simulating third body wear with particles

**Fig. 5:**
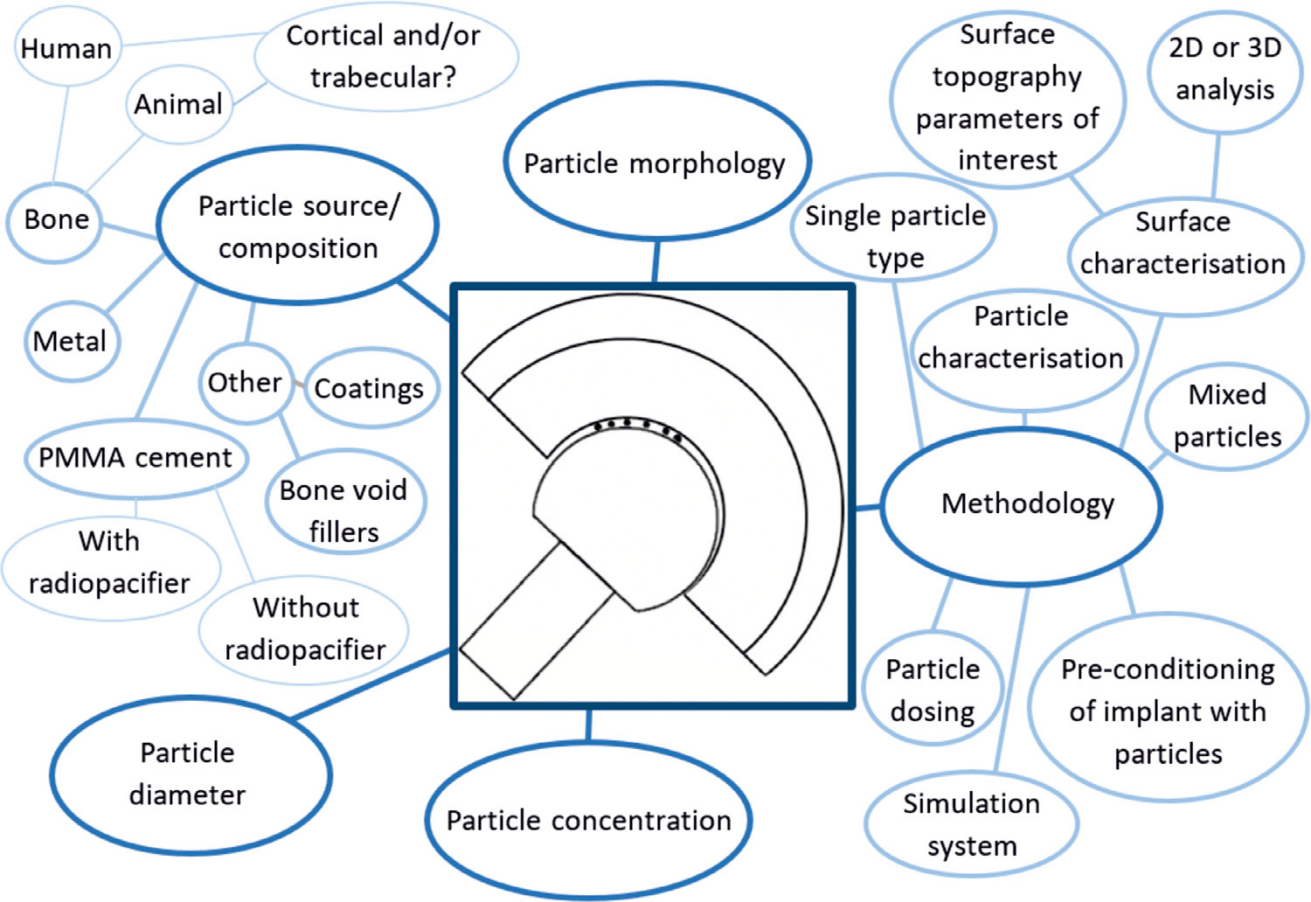
Schematic showing variables which may influence wear when using the particle approach.

When third body particles are added to the lubricant during wear simulation, damage and wear of the articulating surfaces is carried out simultaneously. Whilst this method may replicate the clinical scenario, there are a number of variables to consider which should primarily be decided depending on the research question to be answered. These are summarized in Fig. 5 and include, the particle composition, size, concentration and morphology as well as challenges relating to the methodology which have been approached differently by different research groups (Table S1 a, b and c [43]).

#### 3.1.1 Source of particles

**Fig. 6:**
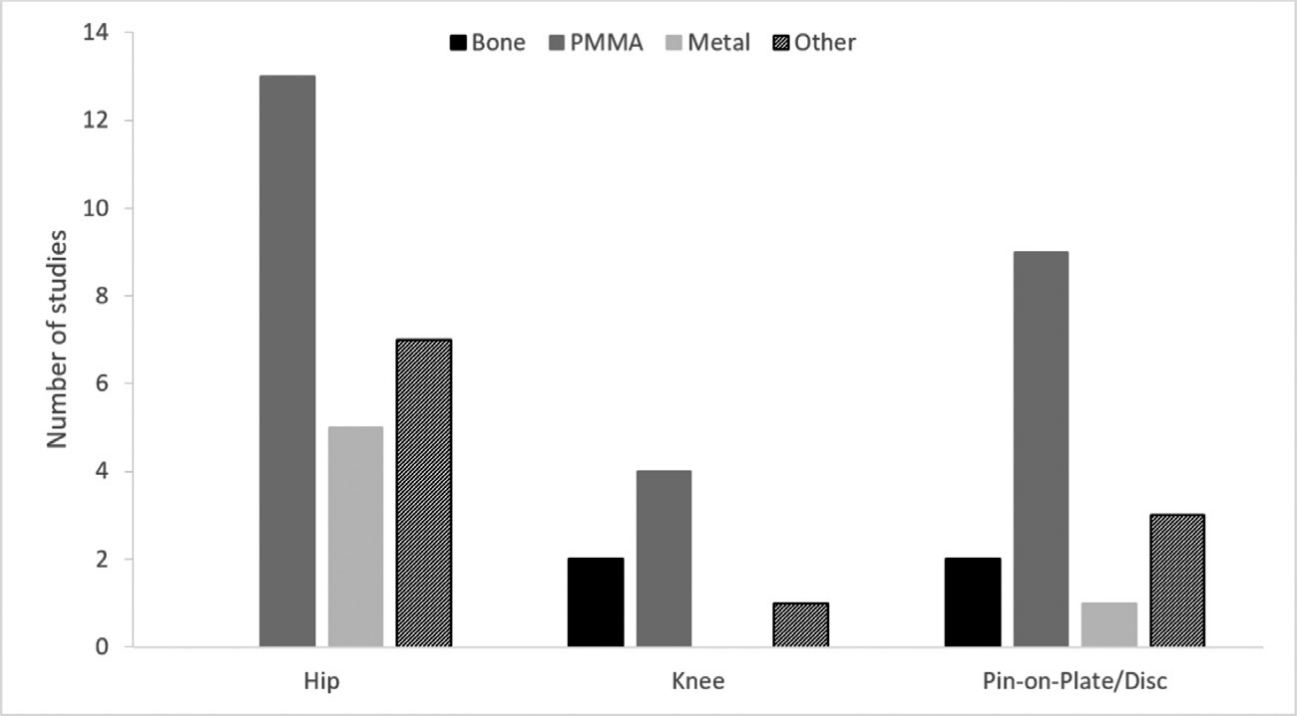
The number of studies using bone, PMMA and metal particles in third body wear simulation; other includes particles such as bone void fillers and hydroxyapatite. [15,23,28,29,33,39,42,45,52,54,55,59,61,62, [70], [71], [72],[74], [75], [76], [77], [78], [79], [80], [81],[83], [84], [85],^87^,^89^,^90^,^92^,^93^].

The particle source for third body wear simulation should be primarily dependint on the research question to be answered. The types of particles that may be present close to a joint replacement include bone, PMMA cement, metal particles originating from either the implant or surgical equipment, coating materials such as hydroxyapatite or metal beads from the implant fixation surface, or other loose materials used close to the articulating surfaces of an implant such as bone void fillers. Rationales as to the materials used include analysis of the debris in fluid from lavage taken during the procedure [35,36] from the damage seen on implants, [74] from particles embedded in polyethylene, [5] from coatings such as hydroxyapatite, [17] from implant fracture, from analysis of contaminants identified in the joint during revision surgery [35,36] and considering the materials used in the implant system [39]. The source of particles used in previous third body wear simulation studies of hip, knee and pin-on-plate is shown in Fig. 6. PMMA particles have most commonly been used; despite the high prevalence of bone particles within the joint following surgery, fewer studies have investigated these particles. Although in a clinical scenario there is potential for particles from a range of sources, the majority of researchers have investigated a single particle source and in some cases have tested multiple particle types on the same implants sequentially [75].

##### 3.1.1.1 Bone fragments/particles

In a study of the fluid from lavage during implantation of total knee replacements, De Baets et al. showed that bone fragments generated by sawing were the most common particle within the joint following the procedure [36]. Bone particles may also originate from detached osteophytes. Third body wear simulation with bone particles has been carried out in both the knee and simple geometry studies using bone sourced from human [15] and animal tissue [75,83]. When added during knee simulation, bone particles have been shown to both deform and become embedded in polyethylene [74,75] however, whilst calcium crystals within the bone are thought to be hard enough to scratch metal surfaces, [94] polyethylene wear was not increased [15,75]. These studies suggest that despite a high likelihood of bone particles being present close to the articulating surfaces of the implant, they may become embedded in and deform the polyethylene, and cause some scratching to metal counterfaces however, the damage may not be of a sufficiently high magnitude to influence polyethylene wear. The use of bone particles experimentally also present difficulties due to inconsistencies between samples, the use of human tissue may present ethical considerations and care should be taken when using animal tissue to match the properties as closely as possible to that of human bone. Therefore, whilst the potential for bone particles to enter the joint space may be high, in a metal-on-polyethylene bearing combination, damage to the metal counterface may not be of sufficiently high magnitude to influence polyethylene wear.

##### 3.1.1.2 PMMA cement particles

PMMA cement particles have been identified within the joint originating either during the initial implantation procedure [36] or following degradation of the cement mantle particularly if the underlying bone begins to resorb and the stress distribution through the implant or fixation changes [95]. PMMA cement is the most commonly used contaminant in third body wear simulation. Trapping unpolymerized PMMA cement power between articulating surfaces has been shown to create minimal damage to metal counterfaces and have no influence on polyethylene wear [15]. Similarly, third body wear simulation with polymerized PMMA cement particles without (radiopaque) additives has also been shown to neither damage metal surfaces nor accelerate polyethylene wear in simple geometry studies [45,52]. The PMMA cement used clinically however, commonly contains radiopaque materials including barium sulphate and zirconium dioxide. When these cements are polymerized, the radiopacifiers agglomerate and the particles formed have a hardness approximately 3 times that of CoCr [62,94]. It is these particles which can scratch metal counterfaces [15]. In simple geometry studies, the use of zirconium dioxide as a radiopacifier has been shown to create more damage to metal counterfaces and to subsequently have a greater effect on wear than barium sulphate, perhaps due to the agglomerates being larger in size [15,45]. Third body wear simulation with PMMA cement particles has also been shown to elevate wear in metal-on-polyethylene hip [39,42,54,55,59,70] and knee implants [75,77,83]. Ceramic femoral heads have been shown to be more scratch resistant than metal heads, [59,70] and in some cases, third body wear simulation of ceramic-on-polyethylene implants resulted in a similar wear rate to that of smooth controls [54,70]. In metal-on-metal articulations, the addition of PMMA cement particles to the articulation has less influence on wear of the implant than introducing harder metal particles into the articulating surfaces [81] and there is some evidence of the PMMA particles being flattened during simulation. Using cement particles to simulate third body wear may only be relevant to cemented implants and when selecting the PMMA cement particles to be used, the cement which is intended to be used for fixation of the implant should be investigated. Third body wear simulation with polymerized PMMA cement particles with a radiopacifier such as zirconium dioxide may elevate polyethylene wear in a metal-on-polyethylene bearing couple particularly whilst the particles are *in situ*; for ceramic-on-polyethylene, ceramic-on-ceramic and metal-on-metal implants, an elevated wear rate has not been seen.

##### 3.1.1.3 Metal particles

Metal particles have been seen embedded in the polyethylene of hip and knee implants. In a study of retrieved knee implants by Que and Topoleski, metal beads derived from the porous coating on the implant fixation surface were observed embedded in almost 20% of UHMWPE tibial components with between 3 and 12 beads in each implant [52]. Similar findings have been seen in retrieved dual mobility hip implants where embedded debris was seen in the inner surface of the liner in 66% of the 56 implants studied. EDS analysis of the particles showed peaks for elements used in the titanium alloy shell (including titanium, aluminium and vanadium); in one sample where severe third body abrasion occurred, traces of cobalt and chromium were present likely originating from the femoral head however, no cobalt or chromium debris was observed [26]. Despite these observations, the occurrence of these particles in the joint following surgery is generally thought to be much lower than that of bone and PMMA cement with < 2% of particles retrieved from the knee implantation site being metallic [36]. Particles such as titanium or stainless steel may originate from surgical tools during the implantation procedure, or perhaps more likely following an extended duration of implantation which may include release from modular interfaces, [29] from the articulating surfaces of the implants if metal-on-metal contact occurs [39] or from the fixation surfaces such as porous coating beads [38]. In metal-on-metal hip replacements, when cobalt chrome beads or titanium particles were trapped between articulating surfaces of a joint, scratches were observed in the articulating surfaces and elevated implant wear measured [16,28,81]. In metal-on-polyethylene hips, introducing titanium particles between articulating surfaces led to high polyethylene wear; ceramic surfaces were more abrasion resistant [29]. Metal particles have been shown to be highly abrasive and have the potential to elevate wear in metal-on-polyethylene and metal-on-metal implants.

##### 3.1.1.4 Other particles

Any loose material implanted close to the articulating surfaces of a joint replacement has potential to migrate between the articulating surfaces of a joint, whether these particles damage the articulating surfaces and accelerate wear should therefore be investigated. For example, bone void fillers used for dead space management in infected arthroplasty revision surgery could enter the joint space, their influence as a third body particle has therefore been investigated in simple geometry, [84,87] hip simulation [78] and knee simulation [90]. Whilst the particles were in suspension in the lubricant, an elevated wear rate of UHMWPE was observed [78,90] however, due to the resorbable nature of the materials, this period of high wear would be short in duration and analysis of the metal surfaces showed the damage created to be below a threshold to influence wear in the longer term [78,90]. Other particles investigated include hydroxyapatite particles, [17] which may originate from coatings on the fixation surfaces of hip and knee implants. Simulation with these particles has been shown to elevate the wear of metal-on-metal hip replacements but to have no influence on the wear of ceramic-on-metal hip replacements [72]. Aluminium oxide particles (alumina) of different sizes are commonly used in polishing applications and have been used in third body wear simulation. Small (1–30 µm) particles of alumina have been used as a surrogate both for the radiopacifiers or the agglomerations they form in PMMA cement and for the hard oxide layer of a metal alloy; larger alumina particles (up to 111 µm diameter) have been used to replicate the ceramic particles which may be released following fracture of a ceramic implant [61,92]. Whilst it is acknowledged that the likelihood of the presence of this type of particle entering the articulating surfaces is low, they have been shown to be highly abrasive, therefore, the consequences of such an abrasive particle entering the joint space may be high implant wear [29,39]. Barium sulfate particles, representing the radiopaque portion of PMMA cement have also been investigated, whilst they abrade metal surfaces and may accelerate wear, the particles are likely to be incorporated into the PMMA cement and therefore it seems more appropriate to study the barium sulfate in the cement [61]. The use of surrogate particles which are commercially available and have a consistent size and composition may be beneficial when considering the particles for use in a standard as commercially available particles may help to reduce interlaboratory variability.

#### 3.1.2 Particle diameter

**Fig. 7:**
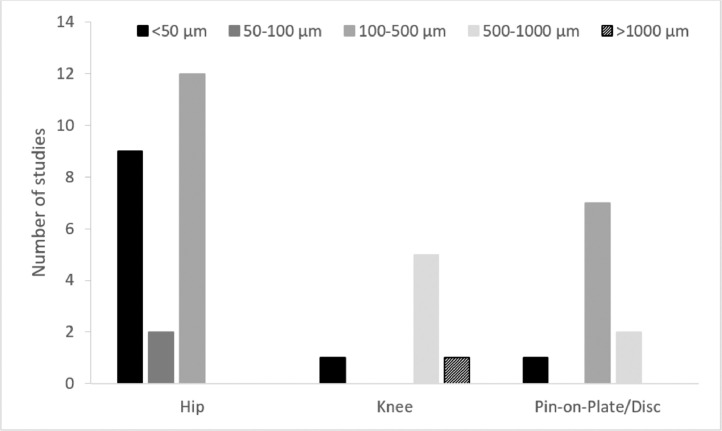
The number of studies where particle diameter has been specified, these have been categorised at < 50 µm, 50–100 µm, 100–500 µm, 500–1000 µm and > 1000 µm particle size used in each study. Note: not all studies have characterised the particles used in wear simulation and where a particle size range has been given, the largest diameter has been taken. [23,28,29,33,39,42,45,54,55,59,61,62,70,72,74,75,[77], [78], [79], [80], [81], [83], [84], [85],^89^,^93^].

The mean size of particles removed from the joint by lavage during the implantation procedure is typically in the range of 200–300 µm diameter with less than 5% of particles recovered being larger than 1 mm [35]. However, whether lavage is more efficient at removing particles of a given diameter is unknown and the size of particles which remain in the joint following implantation is also unknown. The diameter of the particles produced after an extended duration of implantation, such as those occurring due to degeneration of the cement mantle is poorly understood, characterizing the particles embedded in the polyethylene of retrieved implants may give further insight. Investigations of explanted hip components have shown particles to be mainly < 1 mm diameter [23] with Lunberg et al. further reporting the size of embedded particles in a range of 50–500 µm diameter [18]. It is however unclear whether when embedded in the polyethylene degeneration of the particles occurs as the implant articulates. Hydroxyapatite particles originating from the fixation surface of metal shells when embedded in the articulating surface of retrieved implants have been shown to have a smaller diameter than those originally used on the fixation surface [17]. In wear simulation, the particle size is often controlled within a range using sieves, [23,39] a variety of particle sizes have been used in studies carried out to date, however, no parametric studies investigating the influence of particle size on damage or wear have been undertaken. The particle size range that has been used in wear simulation is shown in Fig. 7. In the hip, all the particles used were < 500 µm diameter; in the knee and simple geometry (pin-on-plate/disc) studies, larger particles have more frequently been used. Relatively large bone particles/fragments (up to 2 mm diameter) have been shown to replicate the deformation seen on UHMWPE tibial inserts of knee replacements [74]. Small PMMA cement particles < 30 µm diameter [39, 42,77] as well as larger particles (>500 µm) [54,59,70,75,83] have been shown to elevate wear of UHMWPE in metal-on-polyethylene hip and knee implants but the relationship between particle size and wear is unclear. Other abrasive particles such as alumina particles < 1 µm diameter have also been shown to accelerate wear [39,79,80]. When similar size particles of bone and PMMA cement at similar concentration have been used, an increase in polyethylene wear was observed with PMMA but not with bone [75]. This may suggest that in the laboratory, the size of the particle is less important than its hardness/composition, its concentration and how it is delivered to the simulation system. It is unknown whether the particle size changes over the duration of the study. The most clinically relevant particle size is unclear and may depend on the research question.

#### 3.1.3 Particle concentration

The concentration of particles left in the implantation site following surgery has not been extensively studied, Niki et al. analyzed the particles in the fluid used when washing out the implantation site following total knee replacement surgery. After flushing the site with 4 l of saline, on average 20 PMMA particles and 200 bone particles were isolated per litre saline [35] but the number of particles remaining in the joint, whether the number of particles is consistent between surgeons and procedures is unknown. When carrying out wear simulation, it is important to be mindful that the volume of the lubricant in the test cell is much larger than that of the synovial fluid surrounding the joint and when carrying out wear simulation, the concentration of particles in the lubricant must be sufficiently high to ensure particles become entrained between the articulating surfaces of the joint. It has been suggested that circulation of the lubricant was necessary to keep the particles in suspension, reducing potential sedimentation in the bottom of the test cell and increasing potential for particles to enter the joint [61] however whether this is necessary may depend on the simulation system. The concentration of particles used in wear simulation studies has varied from 0.15 to 10 mg/ml [39,54] depending on the rationale for the study. Only one study has carried out a dose-response type investigation with concentrations of PMMA cement particles from 1 to 10 mg/ml. This study showed an excess of 5 mg/ml particle concentration was required to elevate wear for a specific metal-on-polyethylene hip system being tested in an anatomical configuration. However, the particle concentration chosen for any simulation study should depend on a number of factors including, the simulation system being used, the materials being investigated and the third body particles of interest. Further, the volume of material used during a specific procedure could be included as a factor [90].

#### 3.1.4 Dosing of particles

Whether a single dose of particles is used or whether the particles are replenished each time the lubricant is replaced may depend on the particles investigated. For example, if the particles of interest are resorbable, it may be appropriate to use a single dose of particles representing the short duration the particles are within the body [90]. However, the majority of studies aim to replicate wear caused by non-resorbable particles, in this case, it may be more appropriate to replenish the particles when the serum is changed [54] to ensure particles are available within the lubricant to act as third body particles throughout the simulation. Of the studies reviewed, 15 of the 16 hip simulation studies (Table S1a [26]) and 3 of the 4 knee simulation studies replenished particles (Table S2a [92]).

Some studies of metal-on-polyethylene and ceramic-on-polyethylene hip and knee implants, which showed elevated wear when third body damage was carried out with particles, then continued simulation in a clean environment (without the addition of particles) and showed wear to return to baseline levels [59,78,79,90]. In these studies, any damage created on the articulating surfaces was therefore not of sufficient magnitude to continue to increase wear of polyethylene liners or tibial inserts and the elevated wear rates observed whilst the particles were present in the lubricant must be attributed to another factor. In metal-on-polyethylene hips, examination of surfaces following wear simulation with PMMA cement particles before cleaning showed agglomerations of particles on the heads. It may be these agglomerations of particles rather than any change in surface topography that resulted in the higher wear observed. Other factors such as the geometry or the clearance between head and cup may also influence the build-up of agglomerates on the articulating surfaces. After challenging the implants with particles, further information about whether the magnitude of the damage created on the articulating surfaces by the particles may be gained by additional wear simulation in clean lubricant.

#### 3.1.5 Particle shape

In retrieved hip replacements, embedded particles with both an angular and those with a more spherical morphology have been identified [94]. More angular titanium alloy particles create scratches with a greater peak-to-valley height and cause greater polyethylene wear than spherical cobalt chrome particles of similar diameter [28,81]. The shape of particles embedded long-term in retrieved polyethylene components may change over time [25]. The method used to generate the particles may influence their morphology, whether they agglomerate on surfaces, the severity of the damage they cause to counterfaces and subsequent polyethylene wear. Numerous methods have been used to generate PMMA cement particles including grinding in an oscillating mill either in liquid nitrogen, [39,54,75] or at room temperature, [89] using a mortar and pestle, from turnings, [23,33] and by shaving cement particles from a retrieved cemented knee prosthesis [28]. Whichever method is used, it is important to ensure no contaminants are introduced into the particles during preparation. No side-by-side comparison of differently prepared cement particles has been carried out so the optimum preparation technique for cement particles is unknown. In studies using metal or ceramic particles, a commercially available particle is often used the purity and size range of which has been determined by the manufacturer [28,29,61]. Metal particles of different shapes and materials have been investigated in metal-on-metal hips and showed spherical CoCr beads to create a symmetrical u-shaped scratch morphology more akin to ploughing of the surface and angular titanium particles to create a more random scratch morphology. Despite the different scratch morphologies, the surface roughness parameters investigated were unable to differentiate between the magnitudes of the scratches however, implant wear was higher when a more random geometry scratch was created [28,81].

#### 3.1.6 Overcoming challenges with simulating third body wear with particles

Simulating third body wear of joint replacement materials using particles can present a number of challenges, which may vary depending on the joint investigated and the simulation system used. For simple geometry studies and in knee simulation, the low conforming nature of the contact surfaces means there is a high risk that particles will be ejected from the articulation. One method developed to overcome this potential issue involves carrying out the wear simulation in two phases, in the first phase, particles are trapped between the articulating surfaces and the simulation run without lubricant before adding lubricant continuing the wear simulation, this approach has been taken in simple geometry studies, [33,84,93,96] knee [77,90] and hip simulation [61,89]. In the knee, third body wear simulation has been carried out both with and without this first phase and accelerated polyethylene wear has been seen with both techniques [75,77]. Further, in the hip, Morscher et al. compared methods which introduced particles directly into the articulation and those which put the particles in suspension in the lubricant in an anatomical configuration. Both methods resulted in accelerated polyethylene wear, but when particles were added in suspension in the lubricant, there were fewer embedded third body particles in the polyethylene [17]. In third body wear simulation of hip arthroplasty bearing materials, an inverted configuration (with the acetabular cup mounted inferior to the head) to reduce the number of particles being expelled from the articulating surfaces has frequently been used [28,55,70,72,81]. An anatomical configuration of the hip (with the acetabular cup superior to the head) has also been adopted and with the addition of PMMA particles in suspension, UHMWPE wear is accelerated [28,39,42,54,59,76,78,79,81,92]. The anatomical configuration may be more appropriate as the migration and distribution of wear particles and proteins from the articulating surfaces may better replicate the *in vivo* condition. The two simulation systems have been compared in a short term (10 cycles) metal-on-metal study with minimal lubricant (1 ml) lubricant to wet the articulating surfaces. For PMMA cement particles and spherical CoCr debris, the resulting roughness of the metal was similar for the two simulation systems. However, for titanium particles, in the anatomical configuration, the resulting roughness of the articulating surfaces was significantly higher than in an inverted set up [28]. It is not known whether these results would be replicated in a longer-term wear simulation study with a larger volume of lubricant. The use of a pump system to circulate the lubricant has been suggested to be imperative in anatomical hip systems to reduce the settling of particles in the bottom of the test cell, [39,61] it is not known whether this is necessary for the simulation of all joints but in an anatomical hip configuration, the relative position of the entry and exit ports of the lubricant circulation system have been shown to influence particle settling [61].

When carrying out wear simulation with particles particularly in the knee, the damage created on the femoral component is often linear scratches aligned in an anterior-posterior direction, perpendicular to the flexion axis of the simulator [58]. This predominantly linear scratching is not representative of the damage seen in retrieved implants, which as well as having this linear orientation, also have multidirectional scratches and scuffs [22,97]. Nor is the magnitude of the scratching of sufficient severity to replicate the worst damage on explants. This is likely an artefact of the limited motion of the simulator when running a gait cycle and a limitation of adopting a particle based third body wear simulation protocol. A further limitation of using particles to simulate third body wear occurs when one of the articulating surfaces is a relatively soft material such as UHMPWE and there is potential for the particles to become embedded in the articulating surfaces. Whilst not all retrieval or simulation studies have reported particles being embedded in the polyethylene, PMMA has been seen embedded in acetabular cups [55] and tibial components of the knee [77] and bone particles have also been seen embedded in the tibial components of the knee [74,75]. The presence of embedded particles may replicate the *in vivo* scenario [5] and the deformation and damage seen on polyethylene inserts [74] however, embedded particles present a problem if polyethylene wear is to be measured using a gravimetric technique and errors are likely to occur in the measurements. These errors may be overcome using geometric measurement techniques which are commonly used and have been standardized for the hip [98]; such methods exist for knee implants but have been less widely applied [99], [100], [101]. The incorporation of a 12 h acetone cleaning step to dissolve the PMMA particles before weighing has been suggested, [54] other studies describe extended cleaning in an ultrasonic bath prior to weighing [70]. The potential for loss of accuracy in gravimetric wear measurements when using third body particles should be taken into consideration, as should the potential for a reduction in the reproducibility of the wear and damage of the articulating surfaces and in some cases qualitative assessments of wear may be more appropriate.

#### 3.1.7 Summary

There are many factors to consider when carrying out a third body wear simulation with particles. The relative hardness of the third body particle and the implant counterface is one of the main factors in determining whether wear will be accelerated. It is likely that a higher concentration of particles will increase the chance of particles becoming entrained between the articulating surfaces resulting in higher wear rates. Insufficient studies have been carried out to fully understand how variables such as particle morphology or size influence wear and there is currently insufficient data to understand the wear of all possible bearing materials challenged with all potential third body particles. When determining the conditions to use in a simulation study, without extensive parameterized studies to understand how each variable (particle composition, diameter, morphology, concentration, etc.) influences wear of different bearing materials and different joints, the study design should depend on the research question or risk analysis for the implant.

### 3.2 Approach 2: directly creating third body damage

**Fig. 8:**
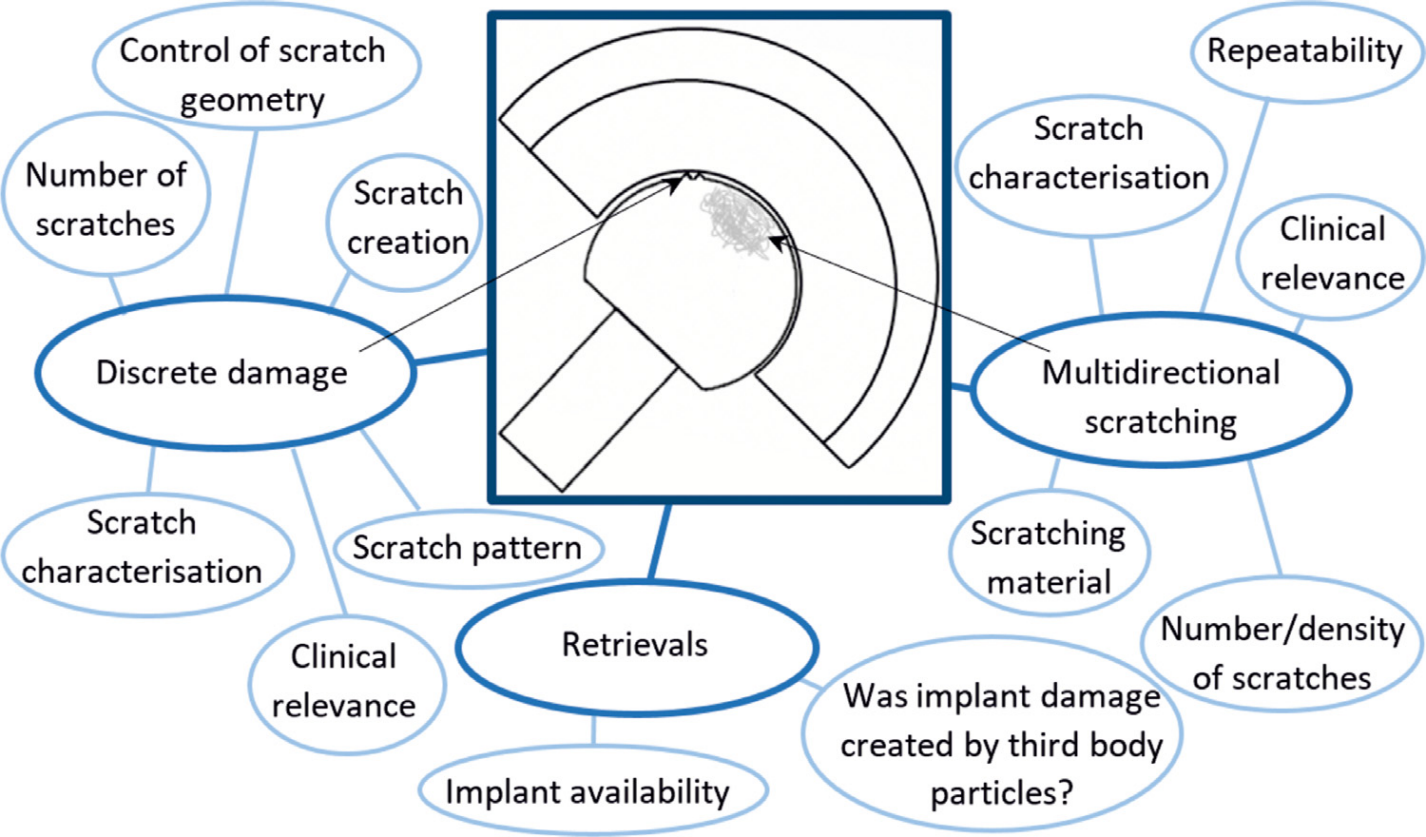
Parameters to consider when carrying out third body damage simulation by directly scratching the counterface or when wear simulation is carried out against retrieved implants to simulate third body wear.

Third body damage can also be simulated by creating clinically relevant damage on the counterface, then carrying out wear simulation against the damaged surfaces. Scratching the implant directly may be advantageous as the damage can be closely controlled. These highly repeatable techniques may reduce the variability of subsequent wear simulation and the damage and wear simulation can be carried out in 2 phases which can give extra information relating to the scratch resistance of materials. In addition, there is no risk of contamination of UHMWPE by particles, which improves the accuracy of assessment of polyethylene wear by gravimetric analysis. A number of approaches have been taken to create this damage informed by the location, magnitude and geometry of scratches on retrieved implants (Table S2 a, b and c [30]). The methods used have been divided into those which create discrete damage in a specific location on the device and those which generate multidirectional scratches on the surface with a more random orientation and morphology. There are several parameters which should be considered when designing a method for creating third body damage on a counterface, these are summarized in Fig. 8.

#### 3.2.1 Methods which create discrete damage

Scratches and scrapes can be inflicted on the surface of materials such as CoCr, ceramic and PEEK using techniques such as a CNC milling machine [86] or a diamond stylus [46,50] to closely control the location and the geometry of the scratch profile. When scratches and scrapes of different magnitude were created on retrieved hip implants, ‘severe’ scratches were shown to elevate polyethylene wear to a greater extent than ‘severe’ scrapes and both scratching and scraping or a combination of the two damage modes could be used to elevate polyethylene wear [27,86]. Creating damage on surfaces using a diamond stylus aims to replicate the most severe scratches caused by entrapment of hard particles between the articulating surfaces and the scratch morphology can be closely controlled similar to that seen in retrieval studies [23,31]. Not all materials behave the same and one of the advantages of using closely controlled methods to create discrete scratches is when investigating new bearing material combinations. For example, when metal surfaces are scratched, the ploughing mechanism produces lips which lead to accelerated wear of polyethylene. In ceramics however, plastic deformation of the material does not occur when scratched. Thus scratches tend not to create lips, and even severe, deep scratches tend not to elevate polyethylene wear [4,23]. Scratches in PEEK form a similar scratch geometry to that of CoCr however, when articulated against polyethylene, a polishing effect of the PEEK is seen and the wear against scratched counterfaces is similar to controls [33]. The number, location and angle of the scratches [46,69] with respect to the direction of the wear simulation have all been shown to influence the wear of polyethylene. The use of a diamond stylus to create scratches has been applied to simple geometry studies, [[31], [32], [33],^46^,^47^,^66^,^69^] hip [50,56,64] and knee [90] wear simulation. Discrete scratches in femoral heads of total hip replacements have also been created by embedding a CoCr bead in polyethylene then articulating this against a femoral head [50,73]. This method allows close control of the location of the damage with the scratch orientation and morphology more representative of the damage seen *in vivo* [51] however, the damage created on CoCr is often less severe (Ra 0.02 µm) than using a diamond stylus (Ra 0.04 µm) and subsequently results in a lower wear of polyethylene [50].

#### 3.2.2 Methods which create multidirectional scratching

Methods have also been used that roughen the hard surfaces of an implant by creating a high number of scratches in multidirectional orientations similar to the scuffs seen on retrieved hip and knee implants. Techniques used include abrading the implant surface using silicon carbide or emery paper, [48,49,53,69] by tumbling in an abrasive material such as a bauxite/alumina media [57,58,60,67,68] or pressing and manipulating the implant in abrasive beads [76]. Using silicon carbide paper to roughen femoral heads allows some control of the orientation of the scratches. On CoCr counterfaces, following treatment with silicone carbide or emery paper, an order of magnitude increase in the mean surface roughness of CoCr heads from approximately 0.02 µm to 0.4 µm [49,53] or even up to 0.8 µm has been measured [48,49] and polyethylene wear has been shown to increase up to 8-fold compared to smooth controls. Another method used to generate random scratches is by tumbling the implants in an abrasive material, this results in a high number of multidirectional scratches being created over the entire surface of the implant. This technique has been applied to both hip [65,68] and knee implants [57,58,60,67] and has shown an order of magnitude increase in mean surface roughness of CoCr resulting in a subsequent increase in wear of polyethylene compared to smooth controls. This technique can be sufficiently controlled to discriminate between implant materials with harder implants such as ceramic [68] or oxidized zirconium components [67] having a lower increase in mean surface roughness and subsequent polyethylene wear than metal components. A more controlled method of generating random scratches has also been described whereby CoCr heads were pressed into a container of abrasive beads then articulated within the beads, to produce a high number of multidirectional scratches over the implant surface. The implants had a higher rate of polyethylene wear than smooth controls however, surface characterization is unknown and to date only descriptive results from metal counterfaces have been reported [76].

Methods which generate numerous random multidirectional scratches over the entire implant surface can replicate the scuffing seen on retrieved implants and, if a sufficient number of scratches are generated, the increase in the surface topography can be consistent over a large number of samples. However, the high number of scratches may make analysis of the surfaces more challenging than when discrete scratches are created and as previous simple geometry studies have shown that scratches in metal-on-polyethylene with a small lip height (< 0.5 µm) to have little influence on wear, it is unlikely that the small scratches will contribute to accelerated wear but may make analysis of the articulating surfaces more difficult [27]. Using these techniques, the magnitude and location of the damage may be more difficult to control than using a diamond stylus, and may not represent the most severe scratching caused by third body particles.

#### 3.2.3 Summary

There are a number of advantages of recreating third body wear by scratching a counterface directly. The methods are generally easier to control than particle based methods with fewer variables to consider, the results give information about the scratch resistance of the counterfaces as well as the wear of materials against the damaged counterfaces without contamination of the polyethylene.

### 3.3 Retrieved samples

Wear simulation against retrieved implants gives possibly the most clinically relevant method to understand how changes in the implant surface topography influence polyethylene wear however, there are a number of challenges associated with using explants. These include, obtaining sufficient numbers of implants with similar changes in surface topography, having appropriate new components with which to pair with the retrieved implants, potential ethical considerations and issues regarding cleaning or handling of the implants. The wear of UHMWPE tibial components against explanted knee femoral components has been investigated. The retrieved femoral components had an initial Ra of 0.1–0.2 µm, an order of magnitude higher than new implants. In wear simulation against the retrieved femoral components, the wear rate was in excess of 3 times higher than against new implants [63]. In the hip, an elevated UHMWPE wear has been demonstrated against retrieved ceramic heads with metal transfer [91] and against scratched oxidized zirconium heads [88]. However, there are issues relating to the selection of the implants which should have ideally been retrieved due to third body wear. In the study of oxidized zirconium heads for example, the damage was created by repeated dislocations rather than third body wear.

## 4 Perspectives and conclusion

This review has emphasized the importance of evaluating implants under a wide range conditions prior to clinical adoption and has discussed methods for simulating third body wear in the laboratory. The methods used can be divided into two groups but whether the method adopted adds particles to the lubricant or scratches the implant surface directly, no method can perfectly replicate all third body wear scenarios seen in retrieved implants and the method or combination of methods used may need to be adapted to accommodate different materials and different joint simulation systems. If establishing a test method, to simulate third body wear with particles, there are a number of variables to consider including the source of particles, their size, morphology and concentration, future parametric research studies could help to improve understanding of how each of these variables influence wear. When carrying out particle methods, different techniques have also been applied such as a two phase approach whereby particles are trapped between the implant surfaces prior to adding the lubricant and starting wear simulation to reduce the chance of particles escaping from between the articulating surfaces and for hips, consideration should be given as to whether the simulation should be carried out in an anatomical or inverted configuration. For scratching methods, creating discrete scratches may replicate the most severe surface damage whilst methods which generate a high number of multidirectional scratches may better replicate scuffing seen on retrieved implants. Hybrids of these techniques may give more clinical relevance, although scratches with a small lip height may not influence wear and may therefore make surface analysis more difficult. When determining the most appropriate methodology to use, the research question to be answered should be the most important consideration for example the introduction of PMMA particles into a cementless implant would have little clinical relevance. More consideration should be given as to whether a standard should give a single protocol under which all implants would be tested, or whether the standard contains guidance on multiple protocols, selection of which should be based on the risk analysis of the device.

## Declaration of Competing Interest

LMJ has received research funding and support in related areas from the following companies: DePuy Synthes, Invibio, Biocomposites, Mathys, and MatOrtho.

## Appendix Supplementary materials

Image, application 1
