# Supplementary material for: Third body damage and wear in arthroplasty bearing materials: A review of laboratory methods
Source: Biomater Biosyst. 2021 Sep 6;4:100028. doi: 10.1016/j.bbiosy.2021.100028 (PMC9934499; doi:10.1016/j.bbiosy.2021.100028)
Supplement: Supplementary file 1 [file mmc1.docx]

**Table S1a: Summary of studies carrying out third body wear simulation with particles in the hip**

*Abbreviations: MOP: Metal-on-polyethylene, XLPE: cross-linked polyethylene, PE: polyethylene, COP: ceramic-on-polyethylene, MOM: Metal-on-metal, COC: ceramic-on-ceramic, COM: ceramic-on-metal, PEEK: polyetheretherketone*

| **Author and year** | **Materials** | **Particle type and diameter (µm)** | **Method** | **Anatomical (A) /Inverted (I)** | **Single or multiple dose of particles** | **Embedded particles?** | **Characterisation of surfaces** | **Wear of materials** |
| --- | --- | --- | --- | --- | --- | --- | --- | --- |
| Affatato et al, 2002[55] | MOP | PMMA with 10% BaSO_4_, 170 µm | Particles added to serum 1 g/l | I | Multiple | Yes | Looping scratches on metal heads | >3-fold increase in wear rate compared to testing in a clean environment |
| Bragdon et al, 2003[39] | MOP | Aluminium oxide particles, 1 µm,  PMMA with BaSO_4_, <30 µm | Particles added to serum (0.15 g/1), maintained in suspension with peristaltic pump | A | Multiple | No | Scuffs and scratches on the metal heads after damage with aluminium oxide, fine scratches with PMMA cement | Increase in wear rate with both particle types, greater increase with aluminium oxide.  Lower wear rate of XLPE than conventional PE |
| Bragdon et al, 2004[61] | MOP | Chromium particles, 1-2 µm  PMMA ± BaSO_4_  BaSO4 particles 1 µm  Al2O3 particles 1 µm | Comparison between particles introduced directly into the articulation (implant assembled inverted with particles in cup then tested anatomically) and particles added to serum | A | Multiple | Yes, when particles introduced directly into articulation | Damage to CoCr heads highest with alumina particles with a dull non-reflective appearance | Wear rates highest with Aluminium oxide particles |
| Bragdon et al, 2005[42] | MOP | PMMA with BaSO_4_, <30 µm | Particles added to serum (0.15 g/l) | A | Multiple |  | Multidirectional scratches on all femoral heads | XLPE more resistant to 3^rd^ body wear than conventional PE |
| De Fine et al, 2021[92] | COC | Ceramic particles (Biolox® Forte (up to 111 µm) or pure Alumina (up to 39 µm)) | Particles added to serum (48 g/l) | A | Multiple |  | Dull/opaque regions on the Biolox® Forte ceramics, no dull regions on the alumina cups | Low wear of ceramics under these third body wear conditions |
| De Villiers et al, 2015[79] | MOP | PMMA + BaSO_4_ 0.3 – 138 µm  Alumina 0.7 – 5.3 µm | Particles added to serum, 5 g/l PMMA  0.15 g/l alumina | A | Multiple, additional testing in clean lubricant | PMMA and alumina particles embedded in metal and PE | Addition of PMMA did not influence R_z_ of metal heads, increase in R_z_ when tested in alumina | Addition of PMMA doubled the wear rate of PE, wear rates returned to baseline levels for 28mm diameter heads when tested in clean lubricant, no such reduction in wear rate with 52mm heads or when alumina used a third body particle. |
| De Villiers et al, 2015[80] | MOP and with and without Chromium Nitride (CrN) coating of metal | Alumina 0.7 – 5.3 µm | 0.15 g/l | A | Multiple, additional testing in clean lubricant | Particles embedded in PE | Scratches 2 µm depth in metal following damage simulation, increase in R_a_ from 0.02 – 0.03 µm, increase in R_z_ from 1.7 – 2.7 µm | Increased wear rate of PE with PMMA, wear rate remained high against scratched CoCr, CrN more scratch resistant than non-coated CoCr. |
| Halim et al, 2014[28] | MOM | CoCr beads (68 – 213 µm), Ti6Al4V particles (175 – 422 µm), PMMA flakes from an explanted knee (58 – 342 µm) | 5 mg particles added to 1 ml lubricant, simulator run for 10 cycles | A and I | Single | No | Abrasion with PMMA less than with metallic particles, lip height measurements an order of magnitude higher with metal particles than PMMA | No wear test |
| Halim et al, 2015[81] | MOM | CoCr beads, Ti6Al4V particles, PMMA flakes as Halim et al[28] | 5 mg of particles added every 0.5 MC | I | Multiple | No | Highest R_a_ when tested with Ti6Al4V particles (159 nm), lower with CoCr (145 nm) and lowest with PMMA (13 nm) | Addition of Ti6AL4V particles led to the highest wear rate (6.4 mm^3^/MC), lower wear with CoCr (4.1 mm^3^/MC) and lowest with PMMA |
| Hembus et al, 2018[89] | MOP (ion treated metal)  COP | PMMA + ZrO_2_  100-200 µm | 5 g/l particles added, 10% particles by weight to the cup and lightly rubbed into the inserts (low force, low motion) | I | Multiple, every 0.5MC | No | Small scratches on the metal and ceramic heads, decrease in R_a_ of metal and ceramic heads compared to controls, no significant difference between metal and ceramic | 5-fold increase in wear rate of polyethylene with third body particles against metal and ceramic heads compared to smooth implants |
| Heuberger et al, 2014[78] | MOP, COC | Calcium sulphate hemihydrate, 0.5-100 µm | 10 g/l calcium sulphate hemihydrate added to lubricant | A | Multiple, additional testing in clean lubricant to simulate wear after bone void filler has resorbed | No, CaSO_4_ is resorbable so not anticipated to become embedded *in vivo* | Scratches observed on the metal heads but no significant difference in Ra between scratched and smooth implants | 39% increase in wear rate of MOP with calcium sulphate in lubricant, wear rates returned to baseline when tested in a clean lubricant). Calcium sulphate had no influence of wear of COC |
| Liao et al, 2010[72] | COM  MOM | HA particles, <100 µm | 0.28 g particles added | I | Multiple, every 0.5 MC |  |  | Addition of particles had no influence on COM wear, wear rate of MOM with particles significantly higher than COM |
| Kubo et al, 2009[70] | MOP  COP | PMMA + 10% BaSO_4_, mean 160 µm, <500 µm | Test run in PMMA slurry (5 g/l) | I | Multiple | PMMA in PE before cleaning, removed by cleaning | Roughness of CoCr and Al_2_O_3_ remained low | PE wear rate increased with PMMA,  COP minimised wear under 3^rd^ body condition |
| Sorimachi et al, 2009[71] | MOP | PMMA | 10 g/l particles | I | Multiple | No | Visible scratching on metal heads after simulation with PMMA | Wear rate of PE increased with PMMA cement |
| Wang and Essner, 2001[54] | MOP  COP | PMMA + 10% BaSO_4_, mean 160 µm, <500 µm | 1-10 g/l PMMA particles | A | Multiple, additional testing carried out in clean lubricant | Acetone used to dissolve PMMA particles |  | For MOP, >5 mg/ml PMMA particles required to elevate wear, PMMA particles had no influence on wear of COP |
| Wang and Schmidig, 2003[59] | MOP  COP | PMMA + 10% BaSO_4_, mean 150 µm, <300 µm | 5 g/l PMMA in lubricant | A | Multiple, additional testing carried out in clean lubricant | Acetone used to dissolve PMMA particles | CoCr heads R_a_ 0.01 µm pre-test, 0.06 µm post-test; ceramic more resistant to scratches than CoCr | Testing with PMMA increased wear rate of MOP (>10x) and COP (3-4x) compared to smooth implants, further testing without PMMA returned wear rates to baseline for MOP and COP |
| Weisenburger et al, 2018[76] | MOP | Pulverised PMMA particles | 0.7 g/l particles | A |  |  |  | No change in wear of PE with addition of PMMA particles |

**Table S1b: Summary of studies carrying out third body wear simulation with particles in the knee**

| **Author and year** | **Materials** | **Particle type and diameter (µm)** | **Method** | **Single or multiple dose of particles** | **Embedded particles?** | **Characterisation of surfaces** | **Wear of materials** |
| --- | --- | --- | --- | --- | --- | --- | --- |
| Cowie et al, 2019[90] | MOP | Calcium sulfate bone void filler crushed *in situ* to a powder | Bone void filler (5cc) added to tibial components, run dry to create damage before adding lubricant | No, single dose of calcium sulfate | No | R_a_ ~0.03 µm, R_p_ ~0.04 µm following damage simulation | Damage created by bone void filler  did not have a lip height of sufficient magnitude to influence wear compared to smooth components |
| Metcalfe et al, 2013[74] | MOP | PMMA 0.5-1 mm, 1-2 mm | Particles added to tibial, run for 100 cycles, lubricated with water | Multiple, 3 sets of 100 cycles | Yes | Polyethylene deformation similar to retrievals, minimal scratching of femoral component | No wear simulation |
| Paulus et al, 2015[83] | MOP UKR | Porcine bone, 672 µm, PMMA + ZrO_2_, 644 µm | 5 g/l debris added to lubricant |  |  |  | 10-fold increase in number of PE particles with PMMA debris compared to bone, no influence on particle size or morphology |
| Schroeder et al, 2013[75] | MOP UKR | Bone, 672 µm, PMMA + ZrO_2_, 644 µm | 5g/l of debris added to lubricant | Multiple | Yes, bone particles | With bone particles, pitting of UHMWPE, with PMMA, abrasion, pitting and scratching | Bone debris had little effect on the wear rate but cement debris led to a significant increase in wear rate |
| Zietz, et al, 2012[77] | MOP  COP | PMMA + ZrO_2_, mean 5 µm, <30 µm | 14 mg of particles added to each knee (7 mg/condyle), first 50 cycles of each 0.5 MC run without lubricant, then lubricant added | Multiple, every 0.5 MC | Yes | Scratches visible on the CoCr femoral components, no scratches on ceramic femoral components | Wear of PE inserts with ceramic femoral components lower than CoCr implants under third-body wear conditions |

**Table S1c: Summary of studies carrying out third body wear simulation with particles in simple geometry pin-on-plate or pin-on-disc**

| **Author and year** | **Materials** | **Particle type and diameter (µm)** | **Method** | **Single or multiple dose of particles** | **Embedded particles?** | **Characterisation of surfaces** | **Wear of materials** |
| --- | --- | --- | --- | --- | --- | --- | --- |
| Caravia et al, 2000[45] | MOP (stainless steel) | PMMA  PMMA + BaSO_4_  PMMA + ZrO_2_  Cancellous bone (bovine)  Cortical bone (bovine), 5-355 µm | Particles added to lubricant 10 g/l | N/A | Yes, particles embedded in UHMWPE in all tests | BaSO_4_ and ZrO_2_ additives in PMMA lead to scratching with ZrO_2_ creating deeper scratches.  Unpolymerised cement did not scratch metal.  Cortical bone creates scratches but smaller than from PMMA with additives | No wear simulation carried out |
| Cooper et al, 1991[23] | COP | PMMA + BaSO_4_  PMMA + ZrO_2_, 5 -500 µm | Smaller particles are more likely to cause damage |  |  | Scratch depth greater with ZiO_2_ cement particles. | No wear simulation carried out |
| Cowie et al 2016[84] | MOP | Bone void fillers, crushed *in situ* to a powder  PMMA (+ BaSO_4_), 500-1000 µm | 2-phases, damage simulation followed by wear testing against damaged surfaces | N/A | No | Following damage simulation, R_a_ <0.02µm on all CoCr plates damaged with bone void fillers and PMMA cement | No significant difference in wear of PE between polished plates and those damaged with particles |
| Cowie et al 2020[33] | UMWPE-on-PEEK and UHMWPE-on-CoCr | PMMA cement (+ ZrO_2_), 500-1000 µm | 2-phases, damage simulation followed by wear testing against damaged surfaces | N/A | No | Following damage simulation, CoCr plates R_a_ ~0.01µm, PEEK plates R_a_ ~0.05µm | Damage simulation had no influence on wear of PE compared to smooth controls, polishing effect of PE against scratched PEEK plates |
| Isaac et al, 1987[15] | MOP (stainless steel) | PMMA + ZrO_2_  PMMA + BaSO_4_  Cadaveric cortical bone | Particles trapped between a loaded UHMWPE pin and a metal plate, plate pulled beneath pin | N/A | Yes | Highest surface roughness with ZrO_2_ particles, more than twice that of BaSO_4_ and cortical bone | No wear simulation carried out |
| Lewicki et al, 2017[87] | MOP | Calcium sulfate bone void filler | Particles trapped between pin and plate | No, single dose to represent bone void filler resorption | Not investigated | Not investigated | BVF did not steady state wear rate in powder or pellet form compared to controls. |
| Manero et al, 2004[62] | MOP (Ti) | PMMA + BaSO_4_  PMMA + ZrO_2_  PMMA + other radiopaque agents, 37-500 µm | 2.5 g cement in 150 ml distilled water (16.6 g/l) |  |  |  | Increased wear in tests run with BaSO_4_ and ZrO_2_ additives. |
| Minakawa et al, 1998[93] | MOP | PMMA cement with different radiopacifiers, bone, <500 µm | Particles trapped between an UHMWPE pin and SS plate, plate pulled beneath pin | N/A | No | Adding radiopacifiers to PMMA increased the number of scratches, more scratches with ZrO_2_ than BaSO_4_ | No wear test carried out |
| Poggie et al, 1994[29] | MOP with TiN and ZrO_2_ coatings  COP | Ti6Al4V, 150-250 µm  Oxidised titanium powder, 1.48 µm | Debris trapped between articulating surfaces. | N/A | Yes | Abrasion resistance of implant materials proportional to surface hardness | Abrasion damage with Ti6Al4V measured geometrically, with TiO_2_, a scoring system was used due to surface contamination |
| Que et al, 2000[52] | MOP | PMMA ± BaSO_4_  Bone | 0.175 g of particles suspended in 4.5 g water (38.8 g/l) |  |  | Scratches on CoCr with bone, PMMA with & without BaSO_4_ | No weight loss of CoCr detected |
| Sagbas et al, 2016[85] | MOP | PMMA spheres, mean 339 nm | 10 g/l in water | N/A | Yes | Pitting of polyethylene and embedded particles | Elevated wear with PMMA particles |

**Table S2a: Summary of studies carrying out third body wear with simulation by creating discrete scratches on the counterfaces in the hip**

*Abbreviations: MOP: Metal-on-polyethylene, XLPE: cross-linked polyethylene, PE: polyethylene, PEEK: polyetheretherketone*

| **Author and date** | **Materials** | **Method** | **Characterisation of surfaces** | **Wear of materials** |
| --- | --- | --- | --- | --- |
| Affatato et al, 2005[64] | MOP | Diamond stylus 35 µm tip used to create 3 scratches in a asterix shape | Pre-test, R_a_ 0.12–0.14 µm, no post-test roughness measurements | XLPE has superior (40x) wear resistance than conventional PE in this study, no control carried out |
| Al-Hajjar et al, 2018[86] | MOP | Scratches and scrapes created in metal heads to represent retrievals | See Kruger et al [27] | Severe scratches led to a higher wear rate of PE than severe scrapes |
| Barbour et al, 2000[50] | MOP | 3 scratches created with a diamond stylus with 25 µm tip and 2.5 N load  3 scratches created with a CoCr bead 250 µm ø embedded in a polyethylene pin 80 N load applied | Stylus R_a_ 0.04 µm  CoCr bead R_a_ 0.02 µm  Scratches with stylus had a higher lip height, were deeper and narrower than with the bead | Scratching with a bead increased wear rate of PE compared to unscratched controls, scratching with a diamond stylus led to a further increase in wear |
| Bowsher and Shelton, 2001[53] | MOP | Metal heads roughened with 400 grit SiC paper to produce overlapping circular scratches | Mean R_a_ 0.4 µm, maximum R_p_ 3 µm | ~8x increase in wear of roughened heads compared to smooth heads under gait conditions |
| Carli et al, 2018[88] | Oxidised zirconium-on-polyethylene and COP | Retrieved femoral heads. Heads classified into either severely damaged (dislocated) or mildly damage (non-dislocated) | Severe scratching on the oxidised zirconium heads which suffered recurrent dislocations, Sa 1.4-8.6 µm; mildly damaged heads with scratches >0.5 cm in length, Sa 0.4-0.7 µm; ceramic heads Sa 0.06-0.2 µm | High PE wear against oxidised zirconium heads which suffered recurrent dislocations; lower wear of PE against ceramic heads with recurrent dislocations similar to oxidised zirconium heads with mild damage |
| Endo et al, 2002[56] | MOP | 3 scratches created on the pole of the femoral heads as Barbour et al [50] | Lip height of scratches, 2-3 µm | Wear rate of PE and XLPE UHMWPE increased 2-3-fold against scratched femoral heads |
| Good et al, 2005[65] | MOP | Tumbling in abrasive media | R_a_ of roughened CoCr heads similar to retrieved samples, R_pm_ of roughened higher than retrieves samples. | With conventional PE, wear rate against roughened heads twice that of smooth implants |
| Heiner et al, 2012[73] | MOP | 5 CoCr beads (300-320 µm ø) embedded and glued in acetabular cup and run for 10,000 cycles under a gait cycle | Circular scratches on femoral heads and acetabular cup. P_p_ of scratches ~0.5 µm. | Wear test not carried out |
| Hembus et al, 2020[91] | COP | Retrieved ceramic heads with metallic transfer | Regions with metallic transfer had a significantly higher surface roughness (R_a_ ~0.35 µm compared to ~0.09 µm in the new components) | Higher wear of UHMWPE liners against heads with metallic transfer than new heads |
| Liao et al, 2008[68] | MOP  MOXLPE  COXLPE | Heads tumbled for 30 minutes with a bauxite/alumina abrasive  media in a table top tumbler | For metal heads, pre tumbling R_a_ ~0.01 µm, R_p_ <0.1 µm, post tumbling R_a_ ~0.04 µm, R_p_ >1.5 µm  For ceramic heads, pre tumbling R_a_ <0.01 µm, R_p_, ~0.05 µm, post tumbling R_a_ <0.01 µm, R_p_ ~0.07 µm | Following scratching, wear of XLPE remained lower than PE, wear of scratched COP similar to smooth implants |
| McKellop et al, 1999[49] | MOP | Femoral heads polished with grit compound or emery paper | Moderately rough – R_a_ 0.4 µm, extremely rough – R_a_ 0.9 µm | Moderate roughening had no influence on PE wear rate compared to polished controls, significant increase in wear rate against extremely rough heads |
| Morrison et al, 2015[82] | MOP and oxidised zirconium on poly | Tumbled in a centrifugal barrel mass-finisher, ~30 s in a 25 µm alumina powder and plastic cone media | After tumbling CoCr implants, peaks formed leading to a positive R_sk_; oxidised zirconium implants had a negative R_sk_ post tumbling. | Tumbling increased the wear rate against CoCr, no increase in wear against oxidised zirconium |
| Wang et al, 1998[48] | MOP | Multidirectional scratches created with SiC paper | R_a_ 0.85 µm | Linear relationship between R_a_ and wear |
| Weisenburger et al 2018[76] | MOP | Femoral heads pressed into abrasive beads, loaded (body weight) then moved through 90° (x10), then repeated in perpendicular direction |  | ~2.5X increase in wear rate compared to smooth controls for conventional PE, 4X increase in wear rate for XLPE |

**Table S2b: Summary of studies carrying out third body wear with simulation by creating discrete scratches on the counterfaces in the knee**

| **Author and date** | **Materials** | **Method** | **Characterisation of surfaces** | **Wear of materials** |
| --- | --- | --- | --- | --- |
| Cowie et al, 2019[90] | MOP | Scratches created with a 200 µm radius stylus in a grid pattern | Scratches with a lip height of >3 µm, R_a_ ~1.3 µm | 7-fold increase in wear rate of PE against scratched tibia components compared to smooth |
| DesJardins et al, 2008[67] | MOP and oxidised zirconium-on-PE | Components roughened by tumbling with 25 µm alumina powder and plastic cone media in a centrifugal finishing barrel for 30s | Following tumbling, increase in R_a_ of femoral components:  CoCr – 0.17 µm  Oxidised zirconia – 0.06 µm | Higher wear against scratched CoCr femoral components than oxidised zirconium |
| Muratoglu et al, 2004[63] | MOP | Explanted femoral components tested against new tibial inserts | Scratches on femoral components primarily orientated in an AP direction, initial R_a_ 0.1-0.2 µm, R_p_ 0.3-0.8 µm | Wear rate of PE ~3.5x higher with explanted femoral components compared to smooth implants |
| Widding et al, 2002[58] | MOP | Femoral components tumbled in a centrifugal mass finisher containing 25 µm alumina and abrasive-embedded plastic cones | Significant increase in R_a_, R_pm_ and R_pk_ over non-tumbled implants |  |
| Widding et al, 2003[60] | MOP | Femoral components tumbled in a centrifugal mass finisher containing 25 µm alumina and abrasive-embedded plastic cones | After tumbling, mean R_a_ 0.1 µm, mean R_pm_ 0.2 µm of femoral components | 3-fold increase in wear rate conventional PE of scratched femoral components compared to smooth |
| Ries et al, 2002[57] | MOP  OxZr-on-PE | Femoral components tumbled with 25 µm alumina powder and plastic cone media in a centrifugal finishing barrel | R_a_ of CoCr femoral components doubled after tumbling, R_pm_ increased 4-fold, no influence of tumbling on R_a_ or R_pm_ of OxZr | Wear of PE 8-fold lower against abraded OxZr compared To CoCr |

**Table S2c: Summary of studies carrying out third body wear with simulation by creating discrete scratches on the counterfaces in simple geometry pin-on-plate or pin-on-disc**

| **Author and date** | **Materials** | **Method** | **Characterisation of surfaces** | **Wear of materials** |
| --- | --- | --- | --- | --- |
| Cowie et al, 2020[33] | PE-on-PEEK and PE-on-CoCr | CoCr and PEEK counterfaces scratched with a (200 µm conical tipped) diamond stylus | Lip height 1, 2 and 4 µm created in PEEK and CoCr | Behaviour of PE-on-PEEK different from PE-on-CoCr, against 4 µm lip height scratches in CoCr, wear of PE significantly higher than against PEEK |
| Dowson et al, 1987[46] | MOP (stainless steel) | Single scratch created either perpendicular to or parallel to wear test with a diamond stylus, plates then lapped to remove lips on scratches, single indentation also investigated | Scratch and indent depth up to 60 µm, lip height up to 36 µm. No change in geometry of scratches after wear simulation (>100 Km sliding distance) | Scratches perpendicular to the wear test have a greater influence (10 x wear rate) on wear than parallel scratches; lapping to remove lips reduced wear rate; single indentation did not influence PE wear |
| Fisher et al, 1995[47] | MOP (stainless steel) | Comparison of pin on plate and pin on disc | Lip height of scratches 1µm, Ra 0.013 µm | 30-fold increase in wear for unidirectional motion tests  70-fold increase in wear for multidirectional motion tests |
| Galvin et al, 2006[66] | MOP | Scratched with a diamond stylus, 100 µm diameter perpendicular to the direction of the wear test | Mean lip height of scratches, 0.8 or 1.8 µm | Increased wear factor with increased lip height of scratches |
| Minakawa et al, 1998[31] | MOP  COP | Scratches created with a diamond stylus, R_p_ 0.1-1 µm | R_p_ of scratches in stainless steel (R_p_ ~1.0 µm) larger than CoCr (R_p_ ~0.4 µm), in ceramic, R_a_ <0.1 µm | Dependence of PE wear on scratch R_p_ or R_pm_ |
| Glennon et al, 2008[69] | MOP | Scratched with 50 µm stylus compared to plates roughened with emery paper | Mean R_a_ 1.2 µm, mean lip height 1.3 µm of scratches. Scratches in different orientations | The direction of the scratches influences PE wear |
| Lancaster et al, 1997[32] | MOP  COP | Lapping, different roughness’s created by different duration of lapping | R_a_ from 0.003-0.010 µm, R_p_ 0.01-0.31 µm | Exponential relationship between surface topography and wear, R_a_ <0.05 µm, diminishing returns in improvement of surface topography reducing wear |
| McNie et al, 2000[51] | MOP | SS bead 10, 150 or 300 µm diameter embedded in polyethylene | Damage created similar to retrieved implants |  |
| Wang et al, 1998[48] | MOP | Scratches created parallel to the direction of motion with SiC paper or alumina paste | R_a_ 0.1-0.7 µm | Exponential relationship between R_a_ and wear, when R_a_ < 0.05 µm, the wear factor is almost independent of surface roughness |
| Weightman and Light, 1986[44] | MOP  COP | Fewer polishing stages adopted to create more rough surfaces | Alumina counterfaces R_a_ up to 0.1 µm, SS counterfaces R_a_ up to 0.75 µm | Exponential increase in wear with R_a_, optimum R_a_ <0.15 µm |
